# Gut microbiota reshapes cancer immunotherapy efficacy: Mechanisms and therapeutic strategies

**DOI:** 10.1002/imt2.156

**Published:** 2024-01-01

**Authors:** Jindong Xie, Manqing Liu, Xinpei Deng, Yuhui Tang, Shaoquan Zheng, Xueqi Ou, Hailin Tang, Xiaoming Xie, Minqing Wu, Yutian Zou

**Affiliations:** ^1^ State Key Laboratory of Oncology in South China, Guangdong Provincial Clinical Research Center for Cancer Sun Yat‐sen University Cancer Center Guangzhou China; ^2^ Hospital of Stomatology, Guanghua School of Stomatology, Guangdong Provincial Key Laboratory of Stomatology Sun Yat‐sen University Guangzhou China; ^3^ Department of Breast Surgery, Breast Disease Center, The First Affiliated Hospital Sun Yat‐sen University Guangzhou China

**Keywords:** cancer, gut microbiota, immunotherapy, immune‐related adverse events, predominant bacteria, precision medicine

## Abstract

Gut microbiota is essential for maintaining local and systemic immune homeostasis in the presence of bacterial challenges. It has been demonstrated that microbiota play contrasting roles in cancer development as well as anticancer immunity. Cancer immunotherapy, a novel anticancer therapy that relies on the stimulation of host immunity, has suffered from a low responding rate and incidence of severe immune‐related adverse events (irAEs). Previous studies have demonstrated that the diversity and composition of gut microbiota were associated with the heterogeneity of therapeutic effects. Therefore, alteration in microbiota taxa can lead to improved clinical outcomes in immunotherapy. In this review, we determine whether microbiota composition or microbiota‐derived metabolites are linked to responses to immunotherapy and irAEs. Moreover, we discuss various approaches to improve immunotherapy efficacy or reduce toxicities by modulating microbiota composition.

## Highlights

- Gut microbiota has been acknowledged as key regulators in host‐mediated anticancer immune response especially during immunotherapy, and antibiotics‐induced dysbiosis often leads to resistance to immunotherapy and poor clinical outcomes.
- Although the mechanisms underlying gut microbiota‐mediated potentiating efficacy while ameliorating side effects of immunotherapy differs across bacteria genus and immunotherapy types, it's generally via enhancing anticancer immunity and remodulating the tumor microenvironment.
- We reviewed the commensal bacteria profiles associated with responders to immunotherapy in various cancer types, characterized by a high diversity with abundance of specific species, which may help predict patients; sensitivity to immunotherapy before treatment.
- Since the causal relationship between individual gut microbiota and response to immunotherapy, we propose gut microbiota act as the future therapeutic target and adjuvant in personalized anticancer regimen, and reviewed the current dilemma and potential strategies to manipulate gut microbiota towards “beneficial bacteria.”

## Graphical abstract

The role of gut microbiota in cancer immunotherapy. Through manipulation of commensals in cancer patients by diet interventions, fecal microbial transplant, prebiotics, probiotics and bacteria consortia, host antitumor immunity can be enhanced by dominance of “beneficial” bacteria in gut lumen and their metabolites. Increased effector T cells and induction of regulatory T cells (Tregs) can be seen in gut‐associated lymphoid tissue, which leads to improved clinical outcomes of cancer immunotherapy with lower incidence of immune‐related adverse events (fecal microbiota transplant; perforin; granzyme B; Treg).

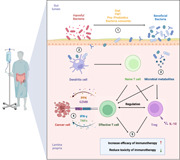

## INTRODUCTION

Globally, multifactorial cancer is the second leading cause of mortality [1], and immune systems are intricately linked to tumor growth [2]. In healthy individuals, abnormal cells can be recognized and removed by immune cells rapidly through immune surveillance. However, cancer cells can escape from host immunity, proliferate and form an intra‐tumoral immunosuppressive environment [3]. Immunotherapy, including adoptive cell therapy (ACT), cancer vaccines, cytokines, oncolytic virus therapies and immune checkpoint inhibitor (ICI) therapy, has dramatically changed the treatment landscape of cancers [4]. Different from conventional anticancer therapies like chemotherapy and radiotherapy, it retards tumor growth indirectly by unleashing and enhancing host Antitumor immune response. Clinical breakthroughs have been achieved through immunotherapy in various tumor types, including hematologic malignancies, melanoma, nonsmall‐cell lung cancer (NSCLC), renal cell cancer (RCC), hepatocellular carcinoma (HCC), and gastrointestinal (GI) cancer [5].

However, immunotherapy is only effective for a small share of patients, and most patients fail immunotherapy due to primary refractoriness and acquired resistance due to the undesired immune infiltrates in tumor microenvironment (TME) [6]. Besides, treatment paradigms are complicated by immune‐related adverse effects, inhibiting the rapid development and clinical application of immunotherapy. Therefore, recent advances in cancer immunotherapy have focused on normalizing the immune defects in TME as well as amplifying antitumor immunity via metabolic reprogramming and combination with other antitumor approaches.

**Figure 1:**
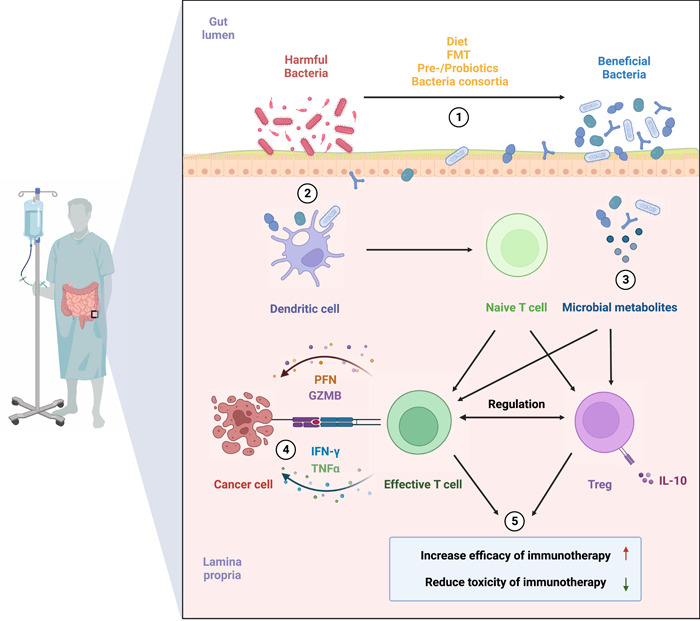
The role of gut microbiota in cancer immunotherapy. Through manipulation of commensals in cancer patients by diet interventions, fecal microbial transplant, prebiotics, probiotics and bacteria consortia, host antitumor immunity can be enhanced by dominance of “beneficial” bacteria in gut lumen and their metabolites. Increased effector T cells and induction of Tregs can be seen in GALT, which leads to improved clinical outcomes of cancer immunotherapy with lower incidence of immune‐related adverse events. FMT, fecal microbiota transplant; GALT, gut‐associated lymphoid tissue; GZMB, granzyme B; IFN‐γ, interferon‐γ; IL‐10, interleukin‐10; PFN, perforin; TNF‐α, tumor necrosis factor‐α; Treg, regulatory T cell.

The commensal microbiota has an intricate relationship between cancer and anticancer therapies in a balance between proinflammation and anti‐inflammation function. Emerging evidence has suggested that gut microbiota and its metabolites can significantly contribute to the efficacy and/or toxicity of immune‐related interventions. Specific microbiota profiling is associated with either improved or defective antitumor immunity between responders and nonresponders. Thus, manipulating gut microbiota towards the dominance of “beneficial” bacteria might be a new therapeutic strategy and a novel biomarker to improve and predict the clinical outcomes of cancer patients receiving immunotherapy (Figure 1).

## THE CANCER‐MICROBIOTA‐IMMUNE AXIS

### The dual role of gut microbiota in cancer

**Figure 2:**
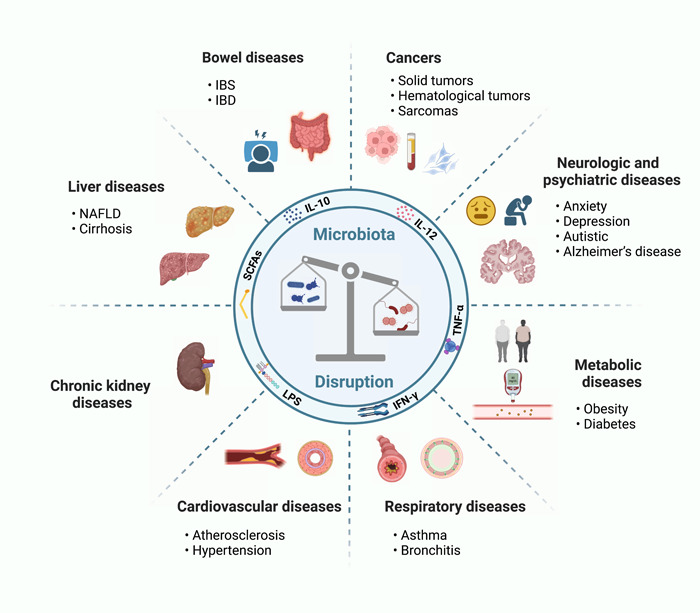
Human microbiota disruption contributes to various diseases including cancers, bowel diseases, liver diseases, chronic kidney diseases, cardiovascular diseases, respiratory diseases, metabolic diseases, and neurologic and psychiatric diseases. IBD, inflammatory bowel disease; IBS, irritable bowel syndrome; IFN‐γ, interferon‐γ; IL‐10, interleukin‐10; IL‐12, interleukin‐12; LPS, lipopolysaccharides; NAFLD, nonalcoholic fatty liver disease; SCFAs, short‐chain fatty acids; TNF‐α, tumor necrosis factor‐α.

Gut microbiota differs widely even among healthy individuals [7]. They reside on intestinal epithelial barriers and are symbiotic with the host. The complex microbial ecosystem is crucial in human health, and disruption of which is associated with chronic inflammation, auto‐immune diseases, heart failure, and even cancer [8, 9] (Figure 2). Previous studies have indicated commensal microbiota plays contrasting roles in cancer initiation, progression, and metastasis.

In 1987, a study observed that patients with chronic infection of *Salmonella typhi* were related to an enhanced risk of death induced by hepatobiliary cancer [10]. In 1994, the World Health Organization designated *Helicobacter pylori* as a class I biological carcinogen and acknowledged it as a crucial pathological factor in gastric adenocarcinoma [11]. Other commensals found in the colon and pancreatic cancers also had the capacity to protumorigenic effects [12]. Researchers have found that the reduction of bacterial load could prominently reduce colorectal tumor growth [13]. Besides these well‐elucidated oncogenic species, disruption of gut microbiota, namely dysbiosis, can also act as a key driver to cancer initiation [14]. The mechanism underlying carcinogenesis caused by gut microbiota includes its producing toxic metabolites, inducing inflammation milieu, and suppressing antitumor immunity, which leads to genomic instability, DNA damage, and immune escape in tumor tissue [15, 16]. Recent studies have envisioned other potential mechanisms, including regulating hormones in circulation or through a reciprocal route called the gut‐brain axis, which further explains how gut microbiota induces dysplasia in distal organs and systems outside the gastrointestinal tract [17].

Conversely, other studies have supported that intact intestinal microbiota served as a protective role against cancer. A causal relationship was observed between antibiotic use and the increased incidence of breast cancer in mice and humans [18, 19]. Iida et al. showed commensal bacteria exert anticancer properties partially through priming tumor‐associated innate myeloid cells for pro‐inflammatory cytokine production, which could alter inflammation at the gut mucous site and in the TME [20]. Through human‐into‐mice fecal microbiota transfer (FMT), Erick et al. found gut microbiota could significantly alleviate pancreatic adenoma (PDAC) tumor growth in mice [21]. These studies suggested that gut microbiota might retard tumor growth partially through altering intratumoral microbial composition and shaping the TME. Interestingly, a recent study showed that gut microbiota and its postbiotics make a contribution to intestinal and immune homeostasis in healthy individuals by modulating function and self‐renewal of intestinal stem cells (ISCs) [22]. Additionally, it can improve immune surveillance through tumor adjuvanticity and antigenicity [23].

Induction of acute inflammatory reactions often stimulates the antitumor immune responses by promoting the maturation of dendritic cells (DC) and effector T cells, and chronic inflammation facilitates tumor progression and treatment resistance [24]. While some bacteria may activate the immune system and thus generate inflammation and oxidative stress, they can also promote tumor development, especially under the context of persistent inflammation, potentially leading to a complex interplay in cancer progression. Infection with *H. pylori* chronically activates disrupted gastric mucosal immune response which leads to accumulation of immunosuppressive cells and finally stomach cancer. Intestinal microbiota or microbial products are essential for inflammatory bowel disease to induce colorectal cancer (CRC), and the use of a common antimicrobial additive may exacerbate colonic inflammation and increase the risk of colitis‐associated colon tumorigenesis in a microbiota‐dependent manner [25, 26]. Further research is needed to fully understand these interactions and elucidate how to adjust the composition of the commensal species to achieve a balance between immune activation and immunosuppression.

### Immunomodulation function of gut microbiota in adaptive and innate cancer immune response

The intricate crosstalk between the commensal microbiota and the host immune system begins at the gut epithelial. Through ligation of pathogen‐associated molecular patterns (PAMPs) derived from gut microbes and pattern recognition receptors (PRRs) presented on a variety of intestinal epithelial cells and innate immune cells, antigen‐presenting cells like DCs are activated, and pro‐inflammatory cytokines are widely produced. Activation and translocation of DCs from gut‐associated lymphoid tissue (GALT) to mesenteric lymph nodes boost the stimulation of naïve CD4+ and CD8+ T cells. Studies have shed light on how gut microbiota affect the generation, differentiation, and infiltration of adaptive and innate immune cells in tumor tissues [27]. In murine models of CRC and melanoma, oral gavage with commensal *Clostridiales* strains potently induced antitumor immunity via potentiating infiltration and activation of intratumoral CD8+ T cells, with therapeutic efficacy superior to anti‐PD‐1 therapy alone [28]. Commensal microbial community could also positively influence patients' outcomes after tumor resection through activating CD8+ T cells‐dependent antitumor response [29].

Previous studies have stated that microbiota‐derived modulators via PRRs have been linked to cancer initiation and development through the activation of innate immune pathways [30, 31]. However, other studies reported that gut microbiota could also affect innate immunity in a beneficial way against cancer. Gut commensals were observed to enhance antitumor T cell immunity by activating DCs via toll‐like receptor 4 (TLR4) signaling in melanoma mice receiving radiation [32]. Besides, the prevalence of specific gut bacteria induced by vancomycin treatment was also associated with potentiation of cytotoxic T cell response via stimulating interleukin 12 (IL‐12) secretion by CD8α+ DCs in mice models of lung and cervical cancer [33]. Lam et al. proposed that gut microbiota could reprogram innate the immune landscape in TME by skewing intratumoral mononuclear phagocytes (MPs) toward immunostimulatory monocytes and DCs through regulating the natural killer (NK) cell‐DC axis and type I interferon (IFN‐I) signaling. These MPs tended to skew toward protumorigenic macrophages when gut microbiota was depleted [34]. Intact commensal bacteria were also found to support immune surveillance in mice with lung carcinoma partially through enhancing γδT17 cell response [35].

## IMPACT OF THE GUT MICROBIOTA ON CANCER IMMUNOTHERAPY

### Involvement of gut microbiota to cancer immunotherapy

In addition to influencing tumor development, the commensal bacteria can also influence treatment outcomes. Preliminary studies have explained the engagement and contribution of gut microbiota to reprogramming anticancer immune responses, presenting a rationale for a novel field, “oncomicrobiotics,” which is focused on how commensal microbiota influences the host‐cancer equilibrium [36].

Through the gut wall where immune cells are located, the gut microbiota interacts with the immune system, which allows it to affect gut immunity as well as immune responses in distal mucosal sites via circulation and systemic metabolism [37]. Antitumor therapies like chemotherapy, radiotherapy, and immunotherapy can damage the integrity of the physical gut epithelial barrier, causing translocation or accumulation of specific microbiota, which leads to alteration in the constitution of the commensal microbiota. Upon Rag2^–^/^–^γc^–^/^–^ mice receiving total body irradiation (TBI) before ACT, an augmented antitumor immunity was seen due to systemic liberation of Lipopolysaccharides (LPS), a common metabolite produced by Gram‐ bacteria) and microbial translocation permitted by TBI‐induced mucosal barrier injury [32]. In mice treated with antibodies against cytotoxic T lymphocyte‐associated antigen 4 (CTLA‐4), the equilibrium between intraepithelial lymphocytes (IELs) and ileal epithelial cells (IECs) was compromised, resulting in a damaged intestinal tract and accumulation of *Bacteroides fragilis* and *Bacteroides cepacian* (which later found to be associated with preferred clinical outcomes) [38].

Dysbiosis of intestinal contents could also affect the clinical outcome of cancer immunotherapy since gut microbiota and host immunity are mutualistic [39]. It is common practice to prescribe antibiotics to patients undergoing cancer treatments to prevent or alleviate opportunistic infections. Beyond its antibacterial effects, evidence have shown that antibiotics are the most common cause of dysbiosis, leading to a detrimental impact on T cell‐based immunotherapies by changing the composition or decreasing the diversity of the gut microbiota [40]. Perturbations of gut microbiota in mice receiving an antibiotic cocktail (vancomycin, imipenem, and neomycin) lead to impaired function of tumor‐infiltrating myeloid‐derived cells and poorer response to CpG‐oligonucleotide immunotherapy [20]. Administration of broad‐spectrum antibiotics, respectively, in murine models of MCA205 sarcomas, Ret melanoma, and MC38 colon cancer all resulted in the compromised antitumor effect of CTLA‐4‐specific antibodies [38]. Retrospective analyses have been conducted in cancer patients based on observations in these preclinical models to determine whether antibiotic premedication could impact the outcomes of ICI treatment. A recent study showed that malignancy patients receiving ICI had adverse outcomes (objective remission rate [ORR], progression‐free survival [PFS], and overall survival [OS]) related to antibiotics administration [41].

### Relationship between gut microbiota composition and efficacy of immunotherapy

#### ICI therapy

Anti‐programmed cell death protein 1 (PD1)/programmed cell death ligand 1 (PD‐L1) and anti‐CTLA‐4 monoclonal antibodies (mAbs) are currently the most widely used ICI therapies. Despite bringing hope to cancer patients who were defined as uncurable before, current ICI therapies are limited by low initial response rate and a long‐term loss of antitumor efficacy. Many preclinical and clinical studies have suggested that interindividual differences in the composition of the commensal microbiota might account for the significant heterogeneity in the success of ICI treatments. The baseline stool samples collected from patients being treated with mAbs were analyzed using shotgun DNA sequencing, 16S rRNA sequencing, and metabolomics, and studies found that decreased diversity or richness of fecal bacterial composition was correlated with lower response rate and worse patient survival [42]. In addition to the diversity of the gut microbiota, further studies have identified that the accumulation of specific bacteria species or strains in the gut microbiota ecosystem was also associated with higher therapeutic efficacy of immunotherapies and enhanced antitumor T cell immunity.

C57BL/6 mice harboring different commensal microbes from Taconic Farms (TAC) and Jackson Laboratory (JAX) were treated with anti‐PD1 mAb, respectively, and JAX‐fed mice (relatively abundant in *Bifidobacterium*) showed far higher response rate to the treatment. This phenotype could be transferred to TAC‐fed mice by cohousing or fecal transplant [43]. Matson et al. identified that *Bifidobacterium longum*, *Enterococcus faecium*, and *Collinsella aerofaciens* were more prevalent in responding versus nonresponding metastatic melanoma patients receiving anti‐PD‐1 blockade, and FMT from responders to germ‐free (GF) mice bearing melanoma could potently facilitate antitumor immune responses and restore therapeutic efficacy of anti‐PD‐L1 blockade in mice [44], indicating that gut microbiota composition was at least partially involved in the efficacy of ICI therapies. Metastatic melanoma patients with high *Faecalibacterium* abundance were prone to benefit from anti‐PD‐1 and anti‐CTLA‐4 therapies with a significantly prolonged PFS compared to those with the dominance of *Bacteroidales* in the gut microbiota [45, 46]. In patients with NSCLC and RCC, sequencing analysis of feces showed that the prevalence of *Akkermansia muciniphila* was associated with favored clinical responses to ICI therapies [47]. Baseline stool samples of patients with advanced‐stage GI cancer collected before and during anti‐PD‐1/PD‐L1 treatment presented an elevation of the *Prevotella/Bacteroides* ratio in patients with preferred clinical outcomes. Besides, it was shown that a subset of responders was significantly enriched in *Prevotella*, *Ruminococcaceae*, and *Lachnospiraceae genus* [48]. In addition, Combination of anti‐PD‐1‐based immunotherapy with traditional Chinese medicine Gegen Qinlian decoction (GQD) eradicated colorectal cancer in mice by remodeling the gut microbiota portraited by a high abundance of *Bacteroides acidifaciens* and increasing the level of CD8 T cells and IFN‐γ [49].

#### Chimeric antigen receptor T cells (CARTs) and allogeneic hematopoietic cell transplantation (allo‐HCT) therapy

ICI treatment has achieved great success in numerous solid tumor types. However, for hematological malignancies, CARTs targeting CD19 and allo‐HCT have been demonstrated as prototype and innovation of T cell‐based anticancer therapies, respectively [50]. A growing understanding of how gut microbiota composition affects allo‐HCT and CART immunotherapy is emerging. A great diversity of commensal bacteria taxa with the dominance of *Eubacterium limosum* was related to a lower risk of progression and relapse after stem cell transplantation [51], while abundance in *Enterococcus* induced by administration of broad‐spectrum antibiotic was linked with exacerbated graft‐versus‐host disease (GVHD) and unfavorable OS [52]. Smith et al. observed that distinct dominance of gut microbiota before CD19‐CART treatment led to different outcomes in patients with B cell malignancies. *Oscillospiraceae*, *Lachnospiraceae*, and *Ruminococcaceae* were enriched in patients achieving a complete response (CR), while the increased presentation of *Peptostreptococcaceae* was associated with resistance to anti‐CD19 CAR‐T cells [53]. To corroborate the result, a two‐center study was conducted by the same team later, and researchers found that a higher abundance of *Ruminococcus*, along with *Bacteroides* and *Faecalibacterium*, were also correlated with higher efficacy and no toxicity development in CART therapy [54]. Notably, “favorable” gut microbiota identified in CAR‐T therapy such as *Ruminococcaceae*, *Faecalibacterium*, and *Lachnospiraceae*, were consistent with the “beneficial” bacteria capable of promoting the efficacy of ICI treatment. However, specific bacterial taxa of the Bacteroides genus showed increased efficacy of CAR‐T immunotherapy in contrast to its role of reducing anticancer immunity in ICI immunotherapy (Table 1).

**Table 1:** Modulation of specific microbiota taxa in cancer immunotherapy.

Gut microbiota taxa	Model	Immunotherapy	Cancer	Major findings	Author/Year	Ref
*Akkermansia muciniphila*	Human and mice	Anti‐PD‐1/PD‐L1	NSCLC, RCC	(a) Induced DCs to secret IL‐12 (b) Increased accumulation of CXCR3+ CD4+ T cells in the TME	Routy et al., 2018	[47]
*Ruminococcaceae*	Human	Anti‐PD‐1/PD‐L1	GI cancer	Associated with distinct bacterial pathways including fermentation to SCFAs, unsaturated fatty acid biosynthesis, vitamin and starch biosynthesis	Peng et al., 2020	[48]
	Human	Anti‐CD19 CAR‐T	B cell lymphoma and leukemia	Improve antitumor effects of CAR‐T cell infusion	Smith et al., 2018	[53]
*Ruminococcus*	Human	Anti‐CD19 CAR‐T	B cell lymphoma and leukemia	(a) Associated with day 100 complete response (b) Possible mechanism: Increased metabolic pathway like for peptidoglycan biosynthesis	Smith et al., 2022	[54]
*Faecalibacterium*	human	Anti‐PD‐1 and Anti‐CTLA‐4	Melanoma	(a) Increased metabolic pathway like amino acid biosynthesis (b) Increased antigen presentation, and improved CD8+ T cells function in the periphery and the TME	Gopalakrishnan et al., 2018	[46]
	Human	Anti‐CD19 CAR‐T	B cell lymphoma and leukemia	(a) Associated with day 100 complete response (b) Possible mechanism: Increased metabolic pathway like for peptidoglycan biosynthesis	Smith et al., 2022	[54]
*Bifidobacterium*	Mice	Anti‐PD‐1	Melanoma	(a) Oral gavage of *Bifidobacterium* alone had the same clinical outcome as ICI therapy (b) Combination treatment of *Bifidobacterium* supplement and ICI therapy could nearly abolish tumor growth (c) elicited DC maturation, thus improved effector function of tumor‐specific CD8+ T cell	Sivan et al., 2015	[43]
*Bifidobacterium longum*	Human	Anti‐PD‐1	Melanoma	(a) Decreased frequency of Tregs (b) Increased frequency of DCs and enhance T_H_1 immune responses	Matson et al., 2018	[44]
*Prevotella*	Human	Anti‐PD‐1/PD‐L1	GI cancer	(a) Associated with distinct bacterial pathways including fermentation to SCFAs, unsaturated fatty acid biosynthesis, vitamin and starch biosynthesis	Peng et al., 2020	[48]
*Lachnospiraceae*	Human	Anti‐PD‐1/PD‐L1	GI cancer	Associated with distinct bacterial pathways including fermentation to SCFAs, unsaturated fatty acid biosynthesis, vitamin and starch biosynthesis	Peng et al., 2020	[48]
*Lachnospiraceae* *Collinsella aerofaciens*	Human	Anti‐CD19 CAR‐T	B cell malignancies	Improve antitumor effects of CAR‐T cell infusion	Smith et al., 2018	[53]
	Human	Anti‐PD‐1	Melanoma	(a) Decreased frequency of Tregs (b) Increased frequency of DCs and enhance T_H_1 immune responses	Matson et al., 2018	[44]
*Enterococcus faecium*	Human	Anti‐PD‐1	Melanoma	(a) Decreased frequency of Tregs (b) Increased frequency of DCs and enhance T_H_1 immune responses	Matson et al., 2018	[44]
*Oscillospiraceae*	Human	Anti‐CD19 CAR‐T	B cell malignancies	Improve antitumor effects of CAR T cell infusion	Smith et al., 2018	[53]

Abbreviations: CAR‐T, chimeric antigen receptor T‐cell; CTLA‐4, cytotoxic T lymphocyte‐associated antigen 4; DC, dendritic cell; GI cancer, gastrointestinal cancer; ICI, immune checkpoint inhibitor; NSCLC, nonsmall cell lung cancer; PD‐1, programmed cell death protein 1; PD‐L1, programmed cell death ligand 1; RCC, renal cell cancer; SCFAs, short‐chain fatty acids; TME, tumor microenvironment.

### Relationship between gut microbiota composition and immune‐related toxicities

As a novel anticancer agent, immunotherapy can activate the host immunity against cancer and shift the inherent immunosuppression tone in TME. However, the enhanced systemic immune response not only exerts effects on tumor tissues but also on normal tissues, causing colitis, hepatitis, pneumonitis, and GVHD, collectively known as immune‐related adverse events (irAEs) [55]. Searching for an intervention that concurrently improves clinical effects and prevents adverse events of immunotherapy has long been a concerning question. Commensal bacteria are pivotal in alleviating overstimulation of the immune system, which induces immune tolerance by potentiating induction of regulatory T cells (Tregs) at mucosal barrier sites and producing immunomodulatory metabolites into circulation [56]. Gut microbiota can maintain host homeostasis in the gut lumen and the rest. Manipulation of commensals might be the key to the question. The intestinal *Blautia* genus was associated with improved overall patient survival while preventing the incidence of lethal GVHD in patients undergoing allo‐HCT [57]. In a preclinical murine model, changes in gut microbiota composition due to anti‐CTLA‐4 treatment were linked to the severity of intestinal lesions, and recolonization of antibiotics‐treated mice with *Bacteroides cepacia* and *Bacteroides fragilis* was observed to be capable of uncoupling efficacy and toxicity of CTLA‐4 blockade [38]. Two prospective studies of patients with metastatic melanoma reported that the overrepresentation of *Bacteroidetes phylum* in baseline stool samples of patients was related to the resistance to the onset of ipilimumab‐induced colitis [45, 58]. A previous study focused on patients with advanced NSCLC showed that the suppressive role of *Lactobacillaceae*, *Raoultella*, and *Akkermensia* species in the development of irAEs was observed during anti‐PD‐1/PD‐L1 treatment [59]. Interestingly, although capable of attenuating immune‐related toxicities, clinical outcomes of immunotherapies were not improved by the *Bacteroidetes phylum* and *Akkermensia* species.

### The beneficial mechanism mediated by gut microbiota in cancer immunotherapy

The human microbiota produces a wide range of genes, and the microbiota encodes peptides that mimic tumor neoantigens [60]. In preclinical murine models, upon stimulation of antimicrobial antigen immunity, bacterial epitope‐specific T cells expand, enter circulation, and transport into the TME, facilitating immune response at distant sites by producing chemokines, expressing CD40L or cross‐reactivity of tumor antigen‐specific T cells [61]. A number of studies have demonstrated the ability of gut microbes to stimulate systemic innate immune responses via interacting with PRRs that mediate anti‐inflammatory or pro‐immune effects [62]. Upon exposure to PAMP which present on microbial surfaces, Antigen‐presenting cells (APCs) located at the gut mucosa or elsewhere upregulate pro‐inflammatory genes such as tumor necrosis factor‐α (TNF‐α), IL‐12, and IFN in a PRR‐dependent manner, thereby promoting Th1/Tc1 immune responses against cancer. Notably, a recent study has proposed that specific gut bacterial species may also promote antitumor immunity by suppressing the expression of PD‐L2 and its binding partner in T cells [63].

**Figure 3:**
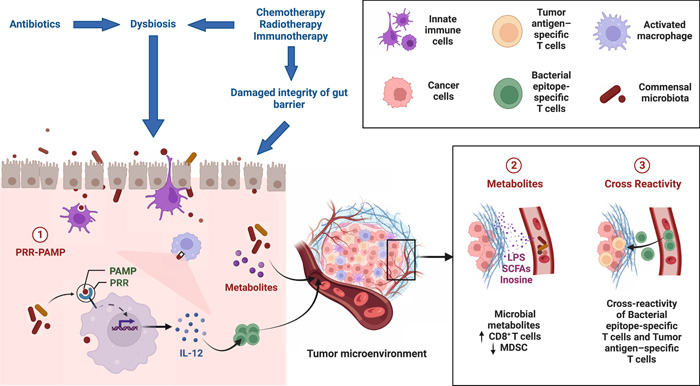
The mechanisms underlying the immunostimulatory involvement of gut microbiota in cancer immunotherapy. Anticancer therapies such as chemotherapy, radiotherapy and immunotherapy can increase permeability of gut epithelial, translocation of bacteria and dysbiosis. During cancer therapy, antibiotics are sometimes used to treat opportunistic infection, which can also lead to disruption of gut microbiota. The mechanism of immunostimulation by gut microbiota includes ligation of PRR and PAMP, release of microbial metabolites such as SCFAs, LPS and inosine, and cross‐reactivity of bacteria epitope‐specific T cells and tumor antigen‐specific T cells. LPS, Lipopolysaccharides; MDSC, myeloid‐derived suppressor cells; PAMP, pathogen‐associated molecular patterns; PRR, pattern recognition receptor; SCFAs, short‐chain fatty acids.

Inosine produced by a subset of intestinal bacteria increases antitumor immunity through enhancing Th1 differentiation and effector function of naïve T cells expressing A2AR [64]. It can also feed effector T cells that infiltrate into TME depletion of glucose by cancer cells [65]. A positive correlation was found between anti‐PD‐1/PD‐L1 responses and bacteria that produce short‐chain fatty acids (SCFAs) like *Eubacterium*, *Lactobacillus*, and *Streptococcus* [48]. Possible mechanisms of immunostimulatory action of gut microbiota in CAR‐T infusion also relied on the microbial metabolites (including bile acid metabolites [66], tryptophan metabolites [67], and SCFAs [68]) and bacterial‐derived membrane fractions (e.g., lipoteichoic acid and LPS) which exert strong influences on T cells via host receptors and other targeted molecules, so hypothetically can modulate anticancer immunity in T‐cell based CAR‐T therapy as well (Figure 3) [50].

## APPLICATIONS OF GUT MICROBIOTA: SERVING AS NEW THERAPEUTIC TOOLS AND BIOMARKERS FOR IMMUNOTHERAPY

### New therapeutic strategies to improve cancer immunotherapy

Cancer patients commonly receive conventional therapies like chemotherapies and radiotherapies before being treated with immunotherapies, which could disrupt gut commensals unfavorably. Thus, manipulating gut microbiota composition to a status of optimal biodiversity and signature before immune‐related interventions might be a new and effective therapeutic tool. Recent studies have raised awareness of the importance of a population‐specific approach to microbiota‐based combination treatments due to the co‐diversification of the gut microbiota with humans [69]. Therefore, interventions will have to be adapted according to the age, lifestyle, comorbidities, comedications, and genetic inheritance of patients for an optimized personal therapy (Table 2).

**Table 2:** Strategies to modulate gut microbiota in cancer immunotherapy and the corresponding clinical trials.

ClinicalTrials.gov Identifier	Study title	Cancer types	Intervention	Objective	Status
NCT04645680	Diet and Immune Effects Trial: DIET—A Randomized Double Blinded Dietary Intervention Study in Patients With Metastatic Melanoma Receiving Immunotherapy	Melanoma	Dietary Intervention: Two different diets vary in fiber content	To assess effect of high‐in‐fiber diet on efficacy and toxicity of immunotherapy drugs pembrolizumab or nivolumab	Recruiting
NCT04866810	The Effect of Diet and Exercise on Immunotherapy and the Microbiome (EDEN)	Melanoma	Dietary Intervention: A plant‐based, high‐fiber diet and a change for high intensity exercise	To see if nutritional intake and physical activity change the gut microbiota in people with melanoma receiving relatlimab and nivolumab immunotherapy	Not Applicable
NCT05251389	FMT to Convert Response to Immunotherapy	Melanoma	Procedure; Fecal Microbial Transplantation	To investigate the influence of FMT on efficacy and toxicity of immunotherapy	Recruiting
NCT04163289	Preventing Toxicity in Renal Cancer Patients Treated With Immunotherapy Using Fecal Microbiota Transplantation (PERFORM)	Renal Cell Carcinoma	Drug: Fecal Microbiota Transplant	To study the safety of FMT combination treatment with ipilimumab and nivolumab, and whether reduce incidence of immune‐related toxicities in patients	Recruiting
NCT04056026	A Single Dose FMT Infusion as an Adjunct to Keytruda for Metastatic Mesothelioma	Mesothelio‐ma	Biological: Fecal Microbiota Transplant	To see if FMT can increase efficacy of an immunotherapy drug Keytruda	Completed
NCT04116775	Fecal Microbiota Transplant and Pembrolizumab for Men With Metastatic Castration Resistant Prostate Cancer	Prostate Cancer	Biological: Fecal Microbiota Transplant	To evaluate anticancer effect of FMT in pembrolizumab treatment	Recruiting
NCT05303493	Camu‐Camu Prebiotic and Immune Checkpoint Inhibition in Patients With Nonsmall Cell Lung Cancer and Melanoma	Advanced NSCLC Stage IV and Melanoma Stage IV	Probiotics drug: Camu Camu Capsules	To evaluate the safety and tolerability of Camu Camu prebiotic in addition to ICI in patients with NSCLC and melanoma	Recruiting
NCT05220124	A Study of Probiotics Administration in the Immunotherapy of Urothelial Bladder Carcinoma	Bladder Urothelial Carcinoma	Probiotics drug: Live Combined (*Bifidobacterium*, *Lac‐tobacillus*, and *Enterococcus Capsules*)	To evaluate clinical benefits of probiotics in immunotherapy	Recruiting
NCT04699721	Clinical Study of Neoadjuvant Chemotherapy and Immunotherapy Combined With Probiotics in Patients With Potential/Resectable NSCLC	Nonsmall Cell Lung Cancer Stage III	Probiotics drug: *Bifidobacterium* trifidum live powder	To evaluate safety and effect of neoadjuvant chemotherapy and immunotherapy combined with probiotics	Recruiting

Abbreviations: CAR‐T, chimeric antigen receptor T‐cell; CTLA‐4, cytotoxic T lymphocyte‐associated antigen 4; DC, dendritic cell; GI cancer, gastrointestinal cancer; ICI, immune checkpoint inhibitor; NSCLC, nonsmall cell lung cancer; PD‐1, programmed cell death protein 1; PD‐L1, programmed cell death ligand 1; RCC, renal cell cancer; SCFAs, short‐chain fatty acids; TME, tumor microenvironment.

#### Dietary intervention

As is known to all, “you are what you eat,” which means long‐term dietary habits affect the composition and activity of microbes residing in the human gut from the scientific aspect. Notably, studies have also indicated that the gut microbiota rapidly respond to dietary changes, even when these alterations occur over a brief period [70]. This underscores the potential of intervening cancer patients' diet as a plausible strategy to suppress tumor growth. Accumulating evidence have shown that patients with a diet high in sugar and fat were correlated with poor response to immunotherapy, while consumption of sufficient dietary fibers and salt led to improved clinical outcomes [71]. Microbial fermentation of dietary fibers produces SCFAs like butyrate and propionate, which can interact with the gut wall and assist in maintaining intestinal immune homeostasis as the main metabolites produced by the gut microbiota of long‐term responders to ICI therapy [72]. High salt condition was associated with significant tumor regression. It enhanced anticancer immunity in tumor‐bearing mice, indicating that salt intake might be a diet‐related factor to affect the efficacy of immunotherapy [73]. Therefore, a diet high in fibers and salt as well as low in fat and sugar can be recommended for cancer patients receiving immunotherapy. A few studies have claimed that distinct diets could result in predictable shifts in existing host bacterial taxa [74]. Higher fiber intake was associated with increased *Provotella*, and higher salt intake led to a lower abundance of *Bacteroides* and *Proteobacteria*, while upregulated *Firmicutes* in a mouse model [75]. Ketogenic diet could upregulate the abundance of commensal *Eisenbergiella massiliensis* which are strongly correlated with the serum concentration of principle ketone body, 3‐hydroxybutyrate (3‐HB) and induce T‐cell based antineoplastic effect in a 3‐HB dependent manner, thereby promoting ICI efficacy and increase overall survival rate in mice with colorectal cancer [76]. For further development of dietary interventions, more studies are needed to determine how dietary interventions can modulate gut microbiota composition and enhance anticancer immune response, as well as the underlying mechanism.

#### Fecal microbiota transplantation (FMT)

By transplanting fecal material from healthy donors to recipients, FMT directly modulates the gut microbiota profiling, which can promote the recolonization of bacteria that exhibit health‐enhancing properties, restore microbial diversity in the gastrointestinal tract, and improve clinical outcomes of immunotherapy [77]. In preclinical models, tumor regression has been demonstrated in mice receiving FMT from patients responding to ICI treatment [78]. In a first‐in‐human clinical trial, the feasibility and safety of FMT treatment in immunotherapy were confirmed with 3 out of 10 patients with anti‐PD‐1‐refractory metastatic melanoma responding to anti‐PD‐1 blockade after FMT from previous responding patients to nonresponding patients, and none of the 10 patients developed severe irAEs during FMT treatment [79]. Another phase I clinical trial enlightened by the first one also focused on the clinical benefit of FMT together with anti–PD‐1 in metastatic melanoma patients who failed immunotherapy, reporting that 2 out of 15 patients achieved partial response while one achieved CR. Despite the fact that all patients in this clinical trial experienced at least one irAE, the degree is minimal [80]. A recent multicenter phase I trial combining healthy donor FMT with the PD‐1 inhibitors showed a more promising result that 13 out of 20 patients with advanced melanoma (previously untreated) responded to the combined treatment including four CR, although 5 out of 20 patients reported grade 3 irAEs during the combination therapy. Safety of FMT as first‐line setting was also ensured for no severe irAEs occurred when FMT was conducted alone. However, it's noteworthy that the similarity between the gut microbiome of donor and recipient only gradually increased over time in responders [81].

#### Defined commensal strains

Besides immune modulation, convenience, and low price, there might be safety risks of transferring chronic diseases like obesity, pathogens, and carcinogenesis during FMT treatment, which makes administering defined commensal strains as exogenous probiotics a preferred alternative for FMT. Developing specific microbiota‐based combinatory treatment has been shown to improve the overall response rate of immunotherapy. Integration of inosine supplementation with checkpoint‐blockade therapy and adoptive T‐cell therapy has led to delayed tumor growth and survival in mice [65], and inosine‐producing microbes like *Bifidobacterium pseudolongum* might represent a novel and efficacious way to deliver the molecule and enhance its accumulation in the TME, which could be added to the diets of cancer patients to improve the efficacy of ICI treatment [82]. Tanoue et al., have identified a rare commensal consortium isolated from healthy human feces, consisting of 11 bacterial strains with 7 *Bacteroidales* and 4 *nonbacteroidales* species, and supplementation with theses 11 strains could enhance both spontaneous and ICI‐mediated antitumour immunity via induction of IFN‐γ‐producing CD8+ T cells in mice intestine without causing colitis [83]. In addition to the colonization of gut with defined commensal species of remarkable clinical benefits in enhancing cancer treatment outcomes, modulation of intestinal bacteria gene circuits yields engineered bacteria, such as *Escherichia coli* (*E. coli*), have recently been presented as innovative anticancer agents by stimulating both innate and adaptive immunity, either alone or as adjuvants when combined with other modalities [84]. Programming immunotherapeutic *Escherichia coli* loaded with anti‐CD47 blocking nanobody could augment activation of intratumoral T cells and effectively shrink tumor size in multiple syngeneic murine tumor models, meanwhile another preclinical study showed engineered *E. coli* Nissle 1917 strain potentiated efficacy of PD‐L1 blockade mediated by increasing L‐ arginine mediated T cells production and activation [85, 86].

#### Prebiotics and probiotics

Prebiotics are nondigestible food ingredients that can serve as nutrients for gut microbes. Studies have investigated that selective enhancement of gut bacteria can improve anticancer therapy outcomes. For example, inulin can stimulate the growth of *Faecalibacterium* and *Bifidobacterium* species which were associated with improved efficacy of immunotherapy before [78]. Novel classes of prebiotics have been discovered to potentiate the antitumor effect of the anti‐PD‐1/anti‐PD‐L1 blockade in mouse models. Ginseng polysaccharides, a main component of *Panax ginseng*, sensitized the tumor to ICI therapy through increasing the microbial metabolites valeric acid and reshaping gut microbial composition from nonresponders towards that of responders in combination with ICI treatment [87]. Diosgenin, a natural steroidal saponin with similar activities to prebiotics, promoted antitumor effects of PD‐1 mAb by modulating intestinal microbiota with upregulation of *Clostridiales*, *Lactobacillus*, and *Sutterella*, and downregulation of *Bacteroides* [88].

Probiotics, including the commonly found *Lactobacillus* and *Bifidobacterium* species, are live microorganisms that are generally acknowledged as a promoter of the host's health in a positive manner [89]. It is reported that cancer patients are prone to self‐administered probiotics as adjuvant to the cancer immunotherapy. Preclinical and clinical studies have reported that probiotic intervention, including *Lactobacillus casei* BL23, *Lactobacillus plantarum* A, or combination of *Bifidobacterium lactis* Bl‐04 and *Lactobacillus acidophilus* NCFM, demonstrated remarkable antitumor immune effect and was capable of restoring the imbalance gut microbial profile [90, 91, 92], which confirmed its potential therapeutic benefits to fight cancer. Furthermore, Sivan et al. found that treating preclinical melanoma with PD‐L1‐specific antibodies while oral gavage with *Bifidobacterium* could nearly abolish tumor growth [43]. Another preclinical research revealed that probiotic *Enterococcus* facilitated the efficacy of checkpoint inhibitor immunotherapy by secreting orthologs of the NlpC/p60 peptidoglycan hydrolase, indicating that screening microbiota species genetically coded with peptidoglycan remodeling activity may obtain potential probiotic candidates capable of enhancing cancer immunotherapy [93]. A recent study reported that oral gavage of a frequently used probiotic *Lactobacillus reuteri* in specific‐pathogen‐free mice challenged with melanoma bolstered the ICI treatment efficacy via promoting the production of probiotic‐released indole‐3‐aldehyde, and such beneficial effect was further recapitulated in melanoma patients [94]. However, there are still safety concerns about over‐the‐counter probiotics use in patients receiving ICI therapy for cancer. On the one hand, probiotic supplements might not be effective if exogenous bacteria colonize poorly in the host intestines and last only a short period. On the other hand, an assessment of commercially available probiotics in murine models of melanoma and CRC showed defective antitumor response to treatment with anti‐PD‐L1 and increased tumor outgrowth [95, 96].

### Novel biomarkers to predict host response to immunotherapy

Previous studies have shown that different fingerprints were detected between responders and nonresponders to ICI therapies based on gut microbiota profiling [14], indicating the potential of utilizing gut microbiota composition and its metabolites to predict the clinical outcomes of immunotherapy. Research have been conducted on gut microbiota as a biomarker for immunotherapy response in recent years, stratifying responders versus nonresponders, as well as predicting the incidence and severity of immune‐related toxicities in a minimal invasive and easy way.

Relative enrichment of certain bacteria species (e.g., *Akkermansia muciniphila* [47], *Bifidobacterium longum* [43], *Bacteroides fragilis* [38], and *Ruminococcaceae* family [45]) was associated with favorable prognosis of PD‐1/PDL‐1/CTLA‐4 inhibitors. Another relative abundance of bacteria (e.g., *Roseburia intestinalis* and *B. thetaiotaomicron* [97]) was reported negatively related to immunotherapy responses. The genetic diversity of bacteria in the gut might serve as an early predictor of the effectiveness of anti‐CD19 CAR T‐cell therapy in patients with large B cell lymphoma, and a high diversity might favor better outcomes [98].

Further study has observed enhanced peptidoglycan biosynthesis in patients who demonstrated a long‐term response to CAR‐T, while upregulated nonoxidative branch of the pentose phosphate pathway was associated with increased incidence of toxicities, indicating metabolites from bacterial taxa might also serve as biomarkers [50]. Therefore, establishing a multi‐parameter model which not only relies on commensal microbial composition and its metabolites level but also takes variables like tumor genomics, comorbidities, age, and germline genetics into consideration might be the future tendency to comprehensively predict whether patients are sensitive to immunotherapies.

## DISCUSSION

Previous studies have shed light on the relationship between the gut, the immune system, the hypothalamic‐pituitary‐adrenal (HPA) axis, and the autonomic nervous system [99]. Consumption of certain food such as dietary fibers will increase the production of short chain fatty acids such as butyrate, which are generated by anaerobic bacteria during fermentation. This will in turn influence the production of neuropeptide such as neuropeptide Y (NPY), and then significantly affect the suppression or activation of certain immune cells [100]. The spleen, a pivotal organ in the immune system, serves as a crucial interface facilitating communication between the brain and the immune system [101]. Use of antibiotic cocktail (ABX) in cancer patients leads to a poor response to T‐cell based immunotherapies via affecting the diversity as well as composition of gut microbiota, but the underlying mechanism remains unclear. Strong correlations were recently observed after a 14‐day ABX treatment between gut microbiota components, spleen weight, splenic cell components and yield of certain compounds in the spleen, brain and plasma, which infers that depletion of microbiota following antibiotics application may affect host immunity through altering the spleen and brain function [102]. More investigations need to be conducted on how gut microbiota influence the immune system through the gut‐brain axis or gut‐microbiota‐spleen‐brain axis.

The vagus nerve, as a major component of the parasympathetic nervous system with dense innervation of the gut, is capable of regulating microbiota‐gut‐brain axis in a bidirectional way [103]. After sensing and transferring the microbiota metabolites information through its afferents to the brain, the vagus nerve fibers subsequently initiate the activation of anti‐inflammatory responses, reduce gut permeability, and thus regulate microbiota composition [104]. Previous studies have also reported that activation of the vagus nerve, namely vagus nerve stimulation (VNS), has potent anti‐inflammatory properties and antitumor effect via triggering antitumor immune cells or upregulating the cholinergic anti‐inflammatory pathway, inferring its potential to establish a favorable intestinal microenvironment for cancer immunotherapy while reducing the occurrence and severity of colitogenic side effects [105, 106, 107]. These findings highlight the importance of VNS in cancer pathology and immunotherapy, suggesting that more investigation should be conducted in the future to unravel the clinical efficacy of a combinatory therapy of VNS and immunotherapy, which may serve as a promising anticancer treatment regimen with the advantages of prescribing less dose of immunotherapeutic drugs to patients, reducing their pain, and suppressing tumor growth effectively in a synergistic way.

While there have been significant research on how the gut dysbiosis can influence the initiation and development of tumors, it is equally important to understand whether intestinal or extraintestinal malignancies promote compositional shifts of the commensal microbiota to their own benefit. During the onset of tumorigenesis, a sustained gut dysbiosis with the hallmark of the *Clostridium* species were recently observed in both human and mice harbored with various cancer types, accompanied with stress ileopathy due to decrease in parasympathetic signaling in the ileal mucosa. Besides, the inhibition of *Clostridium* species by antibiotics vancomycin could overtly prevent cancer‐induced ileopathy, restore intestinal homeostasis and control tumor progression [108]. In addition, whatever anticancer regimens patients received during treatment, such as chemotherapy, radiation therapy, surgery and even immunotherapy itself, could drive a convert on the diversity and profile of microbiota [109], which may in turn influence the efficacy of anticancer therapies. This bidirectional relationship between tumors and the gut microbiota is an intriguing and complex research field, and a broader comprehension on this filed will contribute valuable insights into cancer biology, anticancer treatment strategies and side effect management.

As a modern form of immunotherapy, the combination of cancer vaccines and standard therapies can also enhance antitumor immune responses. However, the unsatisfactory therapeutic efficacy of cancer vaccines in phase III clinical trials has greatly inhibited its development and clinical application. One possible reason is the phenomenon of “immunosenescence” happening in the elderly, who were the majority of cancer patients, suggesting that finding a potent adjuvant in the formulation of cancer vaccines to help restore and stimulate the host immune system is an important tool to improve clinical efficacy. Despite the lack of precise data on cancer vaccines, there has been an apparent correlation between microbiota and vaccination effectiveness against several pathogens, indicating that microbiota might serve as a natural adjuvant to enhance the action of cancer vaccines by providing inflammatory cytokines (IL‐12, IL‐1β, and IFN‐γ) and PAMPs to activate the immune system during vaccination [110, 111]. A study in the murine model has taken one step forward by using microbiota as a real cancer vaccine adjuvant and reported enhanced immune response against tumors [112]. Oncolytic virus, as a new class of immunotherapy agents, has played a pivotal role in cancer treatment by directly killing tumor cells and inducing immunity when used as gene vectors carrying specific checkpoint antibodies. Preclinical studies have shown a plausible relationship between intestinal flora and efficacy of oncolytic virus‐mediated immunotherapy. Yi et al., suggested that gut microbiota may synergistically increase the antitumoral activity of oncolytic virus via the common IFN‐mediated pathway in colorectal cancer [113]. What's more, the antitumor effect of oncolytic adenovirus Ad5D24‐CpG (Ad‐CpG) was recently found to at least partially attribute to gut microbiota, and Bifidobacterium supplementation could hamper tumor progression and intratumoral Treg infiltration in melanoma mice receiving Ad‐CpG treatment [114]. Sarcomas are rare and heterogeneous mesenchymal neoplasms derived from the bone or soft tissues associated with several challenges due to their less immunogenic portrait than other tumor types. Although Immunotherapy has revolutionized cancer treatment, immuno‐oncology agents have not yet been approved for patients with sarcomas attributed to the limitations of low tumor mutation burden and the immunosuppressive TME [115]. However, several research have indicated that immunotherapy may represent an efficient therapeutic strategy for this group of diseases, including cancer vaccines, immune checkpoint blockade and adoptive cell transfer [116]. Future studies investigating novel immunotherapy strategies in rare cancer types like sarcomas should incorporate the analysis and intervention of commensal bacteria, which will allow for a better understanding of the gut microbiota involved in sensitivity and treatment resistance to immune‐oncology agents.

Regarding the preliminary research and clinical intervention of gut microbiota in immunotherapy, there are still some issues to be resolved. For example, current tools to explore the effect of gut microbiota mainly include the antibiotics treatment models or germ‐free (GF) mouse models, among which the GF mice were widely acknowledged as the gold standard due to their complete microbial depletion and ability to be exclusively colonized with defined microbes [117]. However, concerns have been raised regarding the impaired development of the immune system in GF mice due to a lack of early immune education, which potentially impacts their applicability in microbiota studies in terms of immune‐related conditions, including cancer immunotherapy [118]. In addition, GF mouse models are expensive to acquire and maintain, thus antibiotics treatment may present as a cost‐effective and easily accessible alternative to germ‐free models with less limitation. In addition, as for microbiota modulation approaches, the conventional screening measure is unable to detect side effects of FMT [119], necessitating meticulous collection of stools from healthy donors to prevent potential infection caused by transfer of pathogenic bacteria during FMT. Using defined bacterium as daily medication might face technological obstacles like finding optimal culture conditions and encapsulation which allows large‐scale manufacture in vivo and preserved biological activities before it takes effect in our gut.

**Figure 4:**
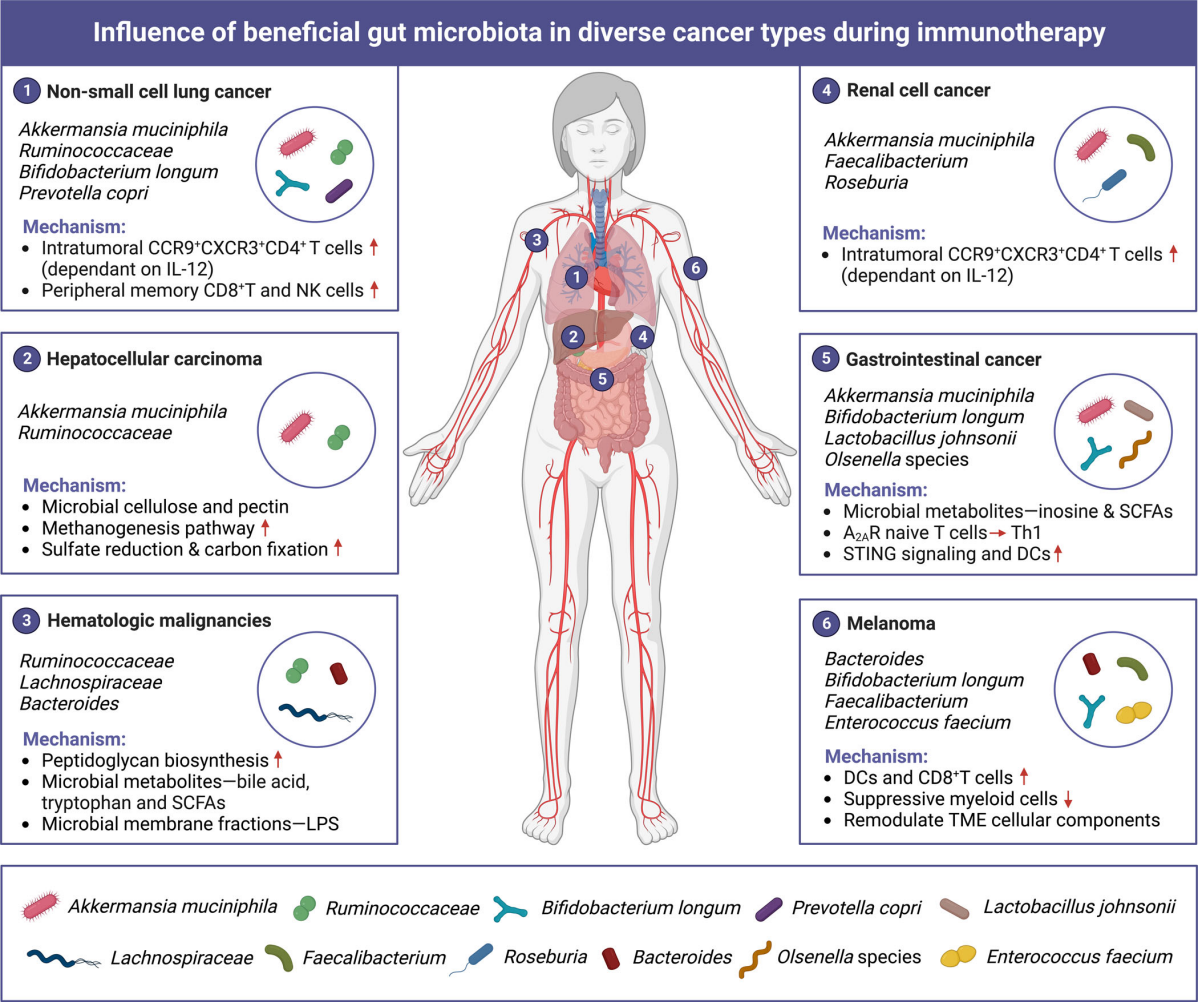
Influence of beneficial gut microbiota in diverse cancer types during immunotherapy. Gut microbiota has contrast role in cancer initiation and development, which not only affects gastrointestinal (GI) cancer locally, but also impacts cancer developed in distal organs including nonsmall‐cell lung cancer, hepatocellular carcinoma, hematologic malignancies, renal cell cancer, and melanoma.

Microbiota effects are not likely dependent on one species and might be caused by diverse microbiota compositions, and future research should focus on identifying a group of microbe consortium with great benefit to optimizing cancer immunotherapy. Comprehensive mapping of the biological effects and modes of action of prebiotics and probiotics for each cancer type still has not been well‐elucidated, which inhibits their application in individual precision medicine (Figure 4). Therefore, the development of cheap, rapid and accurate testing techniques on patient serum and stool samples, which combines meta‐transcriptomic sequencing, metagenomic or metabolomic analysis, will help elucidate the underlying molecular mechanism involved in different therapeutic responses and propose novel therapeutic targets for cancer patients with microbiota‐related immunotherapy resistance.

## CONCLUSION

Gut microbiota serves as pivotal intermediate between the gut, brain, spleen and immune system, maintaining homeostasis of the organic whole. Unfavored intestinal flora leads to intestinal or extraintestinal malignancies, hampers anticancer immunity and participates in shaping the immunosuppressive TME. Accumulating studies have observed that the diversity and composition of host gut microbiota were associated with the efficacy of immunotherapy as well as the incidence of irAEs, which shed lights on utilizing microbiota as novel biomarkers to predict patients' response to immunotherapy and targeting microbiota as potential anticancer agents alone or as adjuvant. This highlights the importance of investigating precise and safe approaches which could alter microbiota profile in cancer patients towards a more diverse composition in favor of “beneficial bacteria.” Meanwhile, more studies are needed to comprehensively mapping the blueprints of beneficial commensal strains across various cancer types and tracking the dynamic change of gut microbiota throughout the treatment course, and focus on the complex role of gut microbiota in new form of immunotherapy in the future.

## AUTHOR CONTRIBUTIONS

Yutian Zou, Minqing Wu, and Xiaoming Xie conceived the idea and edited the manuscript. Jindong Xie, Manqing Liu, and Xinpei Deng collected the data and wrote the manuscript. Jindong Xie and Manqing Liu drew the figures. Yuhui Tang, Shaoquan Zheng, Xueqi Ou, and Hailin Tang validated and supervised the manuscript. All authors have read the final manuscript and approved it for publication.

## CONFLICT OF INTEREST STATEMENT

The authors declare no conflict of interest.

## Data Availability

Supplementary information (graphical abstract, slides, videos, Chinese translated version, and update materials) is available online DOI or http://www.imeta.science/.
